# Influence of S and Mn Initial Concentrations on the Graphite Branching in Gray Cast Iron as Quantified by 2D Image Analysis

**DOI:** 10.3390/ma18214837

**Published:** 2025-10-22

**Authors:** Luis Filiberto De Santiago-Méndez, Manuel de Jesús Castro-Román, Martín Herrera-Trejo, Hector Mancha-Molinar, Beñat Bravo

**Affiliations:** 1 Cinvestav Unidad Saltillo, Av. Industria Metalúrgica 1062, Ramos Arizpe 25900, Coahuila, Mexico; luis.mendez@cinvestav.edu.mx (L.F.D.S.-M.); martin.herrera@cinvestav.edu.mx (M.H.-T.); 2 Independent Researcher, Formerly in TUPY, Blvd. Isidro López Zertuche 4003, Saltillo 25230, Coahuila, Mexico; hmancha53@gmail.com; 3 Azterlan, Basque Research and Technology Alliance, Aliendalde Auzunea 6, 48200 Bizkaia, Spain; bbravo@azterlan.es

**Keywords:** graphite morphology, flakes branching, flakes size, skeletonization technique, ImageJ

## Abstract

The morphology changes in graphite flakes due to the difference in S and Mn contents were analyzed in gray iron samples with a Carbon Equivalent (CE) of 4.0. Although these Mn and S contents are within the range of industrial usage, the morphological characteristics of graphite flakes among the different samples show significant changes in their size and distribution. Graphite flake size was estimated using the Feret diameter, and the flake’s distribution was visually characterized following established standards. As it was observed that graphite flakes also differ in branching, a new procedure was developed to quantify such branching. Based on a skeletonization technique, this new procedure provides data to obtain additional microstructural parameters of the graphite flakes, such as the percentage of branched flakes and the longest shortest path (LSP) of each graphite flake. Microstructural characterization included measuring the eutectic cell count. The results indicate that Feret values and LSP show only weak correlations with concentration estimates from initial S and Mn. The most notable relationships are between sulfur content and Feret or LSP values. In contrast, the branching percentage correlates well with free sulfur at 1150 °C and eutectic cell count and is also linked to graphite distribution types (A or B). Notably, branching percentage offers a straightforward morphological parameter that enhances graphite flake characterization.

## 1. Introduction

Gray cast irons are classified based on their ultimate tensile strength (UTS) according to the A48/A48-22 standard [1]. In the absence of casting defects, UTS values depend only on the microstructural constituents of the irons. This microstructure is generally controlled by the piece’s cooling conditions and the chemical composition of the cast metal. The Carbon Equivalent (CE) predominantly affects the UTS values of gray irons [2], as it determines the amount of proeutectic austenite, which primarily determines the UTS range values. At similar CE values, the UTS increases with the presence of pearlite [2,3]. Promoting the conversion of austenite to pearlite is then suitable, and it is usually performed by adding elements such as Cu and Sn [4,5].

At a given CE and pearlitic or ferritic microstructure, other elements, such as S and Mn, may also significantly affect additional microstructure features of cast irons and, therefore, the properties of gray iron and castings’ soundness. Several microstructural characteristics depend on the S and Mn contents and their interaction; among them are the eventual presence of FeS, MnS, and carbides, the changes in the flake graphite shape, the eutectic cell count, and the matrix structure, as reviewed by Goodrich et al. [6]. The interaction between Mn and S in gray irons has been of great interest from the early 20th century to the present [6,7,8,9,10,11,12,13]. The interaction of Mn-S needs to be revisited because Mn levels have been increasing in cast irons through the recycling of steel with higher Mn concentrations. Consequently, it is necessary to know how a greater concentration of Mn than usual could affect the behavior of the microstructure of cast irons.

The primary goal of adding manganese to cast iron is to counteract the harmful effects of sulfur. Generally, when the Mn/S weight relationship is 1.7, Mn/S is considered a “balanced” one; over 1.7, Mn is in excess, while under 1.7, sulfur is in excess [6,8]. It has been observed that Mn/S > 1.7 promotes the formation of “normal” graphite and ferrite formation and decreases the count of eutectic cells. At the same time, excess sulfur, Mn/S < 1.7, favors the formation of FeS, “spiky” graphite, pearlite, and increases the count of eutectic cells [6]. The FeS can cause hot shortness and embrittlement in iron castings, as reviewed by several authors [8,11,12]. Manganese excess is also needed to reduce the chill depth and overcome the effects of free sulfur in promoting abnormal graphite forms [8]. Based on a review of previous works, Goodrich et al. reported general qualitative trends of the effects of S and Mn on chill and mottle depth, graphite form, matrix structure, and eutectic cell count [6]. However, the statistical correlations between microstructure features and Mn and S content, Mn_ex_, or the Mn/S relationship are generally reported to be low, leading to some discrepancies among the studies.

After examining the structures and the mechanical properties of irons with various compositions, Norbury proposed using excess manganese, wt%Mn_ex_, content over the value required to form MnS with the sulfur in the iron [7]. Equation (1) defines the wt%Mn_ex_.

wt%Mn_ex_ = wt%Mn − 1.7 wt%S, (1)

Norbury proposed a wt%Mn_ex_ value of 0.3, while Mampey [14] suggested using a wt%Mn_ex_ of 0.2–0.3% when sulfur content is ≤0.1 wt% and 0.4–0.5 wt%Mn_ex_ when wt%S ≥ 0.1.

Recently, the dissolved sulfur in the liquid metal at the eutectic reaction temperature was proposed as a criterion to balance the Mn content with the S content present in gray iron [11,12]. They suggested using manganese content that limits the free sulfur content in the liquid during the eutectic reaction to 0.08 wt%, as calculated from a MnS solubility product of 0.03 at 1150 °C. This approach permits estimating the metal’s sulfur content that could interact with graphite during its growth, which is a scientifically based aspect. However, free sulfur can be calculated from the values of wt%Mn_ex_ and vice versa, with conversion values depending on the MnS solubility product value estimated at the eutectic temperature. According to the data from Gundlach et al., a free sulfur content of 0.08 wt% is equivalent to a value of wt%Mn_ex_ = 0.24.

In addition to S and Mn, and other elements, the morphology of graphite is also related to its undercooling growth. In irons with the same composition, this undercooling is typically decreased when the cooling rate is lowered or when graphite inoculation is enhanced. Laboratory studies with high-purity materials have shown that graphite can change its morphology from plates to coral graphite when the cooling rate increases to a critical value, which depends on the sulfur content [15]. At the same cooling rate, plate graphite changes to a lamellar form when undercooling for growth increases, and further changes to undercooled graphite if the sulfur content increases [15].

Only the main qualitative tendencies about relationships between graphite morphology and mechanical properties have been inferred. For example, it has been noted that the quantity, size, morphology, and distribution of graphite flakes are crucial in determining the mechanical behavior of gray cast iron [16,17,18,19]. High UTS has been observed in cast irons with sulfur contents lower than 0.06 wt% [8,11]; it could then be inferred that graphite morphology associated with this sulfur content is suitable for achieving such UTS levels. The morphology of graphite also influences thermal conductivity and heat diffusivity. The conductivity of gray iron depends mainly on its graphite structure and not so much on its matrix structure [20]. The straightest and long graphite flakes improve the heat conduction capacity [21], as does thermal conductivity [22]. Hecht et al. reported that the thermal diffusivity of cast iron has a strong linear correlation to graphite flake length [23].

While the reported main qualitative tendencies of Mn and S’s effects on cast iron’s microstructure and mechanical properties are generally similar, some aspects that allow for better understanding are still to be developed. Some of these aspects involve new parameters, derived from the cast iron composition, that correlate better with the microstructure and mechanical properties. Additionally, new, enhanced, and more accessible techniques and tools to characterize graphite morphology may still be necessary.

New simulation tools could enhance our understanding of the effects of solutes on graphite growth. For example, molecular dynamics simulations have been conducted to evaluate the effect of Mg segregation in crystallographic planes on the growth of nodular graphite [24]. Additionally, ab initio calculations were performed to investigate the possibility of preferential solute positioning on the graphite structure, aiming to understand its degeneracy [15]. These types of studies demonstrate that assessing the preferential positioning of solutes at the growing interfaces of graphite is now possible. These solutal interactions with the growing graphite interface are believed to provoke changes in graphite growth directions and, consequently, graphite’s morphology. When preferred graphite growth directions are assumed, graphite growth models can be developed, as proposed for growing plate-like graphite [25] and nodular graphite [15].

As reviewed here, the morphology of graphite flakes in gray cast irons varies significantly when the concentrations of sulfur and manganese are changed, even when other parameters, such as CE, cooling rate, and melting and inoculation procedures, are kept constant. Furthermore, the morphology of graphite flakes is so varied and complex that only their visual characterization, based on standards such as ASTM A247-19 [26], ISO 945 [27], or an equivalent, has been possible. This visual characterization remains functional when comparing different casting samples, but the information is limited to a quantitative nature. The complex geometry of graphite flakes even complicates the measurement of flake lengths [28], which are often limited to Feret diameter measurements, as seen in [29], or other methods considered in ASTM A247-67 [30]. The Feret diameter has also been used for developing or testing new graphite characterization techniques. Some guidance for performing image analysis measurements is given in ISO 945-2 [31]. However, some other characteristics of graphite flakes that appear attractive have been only visually characterized [32,33] and mentioned as abnormal graphite forms [8,34], normal graphite [35,36], different types of graphite according to ASTM A247, cited by Gundlach et al. [11], or various levels of branching [6,8,37].

However, limitations on graphite morphology characterization could be overcome with the currently available computing and image processing capabilities. Several studies have focused on automated image analysis for characterizing the morphology of graphite [38,39,40,41,42,43]. Additionally, techniques for 3D microstructure analysis are being developed [44,45]. Furthermore, a new method for overviewing patterns of graphite structure in cross-sections was developed [46]. This research was conducted to investigate flake morphological parameters that are currently unquantified but accessible through image analysis, and to compare them with classifications made using standard methods. For image analysis, the free ImageJ software was chosen to characterize graphite flakes in a hypoeutectic cast iron with various sulfur and manganese contents.

## 2. Material and Methods

### 2.1. Preparation of Cast Iron Samples

Twelve alloys of hypoeutectic composition with similar C, Si, Cu, and Sn concentrations but different S and Mn contents were fabricated. Sulfur concentration was fixed at four levels: 0.025, 0.08, 0.12, and 0.15 wt%, and for each sulfur content, three levels of Mn were tested: 0.1, 0.4, and 0.7 wt%. The chemical composition of alloys and Mn_ex_ and free S values are presented in Table 1. Samples were labeled according to their Mn and Sulfur content. Letters L, M, and H correspond to 0.1, 0.4, and 0.7 wt% Mn, respectively. The number following these letters corresponds to the sulfur content.

The Mn_ex_ values shown in Table 1 were calculated based on Equation (1), and the percentage of sulfur dissolved in the liquid metal at 1150 °C, free S, was calculated according to the procedure described in Annex A. The product K_SP_ shown in Table 1 corresponds to the product of the initial contents of S and Mn.

The material was melted in a 100 kg induction furnace (250 Hz, 100 kW). The initial furnace charge consisted of 52 kg of ingot, 43 kg of returns, 1.65 kg of graphite, 1.4 kg of FeSi75, and 0.8 kg of copper. Thermolan^®^, (Azterlan, Vizcaya, Spain), a thermal analysis device, was used to finalize the check of C and Si contents in the liquid metal for its eventual correction. The melting, inoculation, and pouring procedures were kept constant in all experiments.

**Figure 1:**
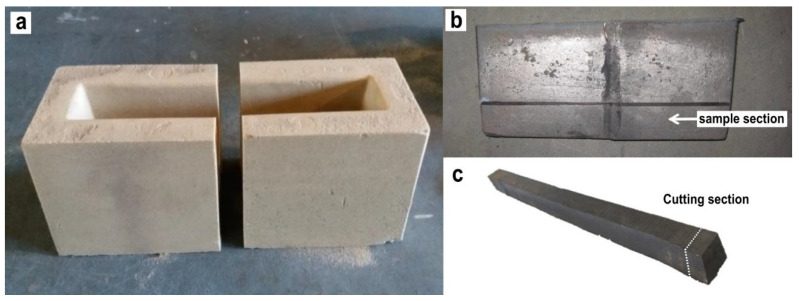
(**a**) II UNE-EN 1563 Keel Blocks, (**b**) Ingot sample without superior part or feeder, (**c**) Cutting section for image analysis in this study.

The alloys were cast in Y-shaped sand molds of type II, UNE-EN 1563 Keel Blocks [47], but modified to have a length of 300 mm (Figure 1a). This length was necessary to obtain samples for subsequent studies. Before pouring, extra-fine Superseed inoculant was added to the mold at a 0.15 wt% of the metal weight of the castings. The cast blocks were left to rest for at least 8 h after they were poured.

The ingot samples were cut into four parts along the longitudinal axis. For the metallographic study, a sample was extracted at the same location in the lower part of each ingot, as schematized in Figure 1c. These samples were prepared by dry grinding with sandpaper from #120 to #1200. Subsequently, the pieces were polished with 3 µm and 1 µm diamond paste.

### 2.2. Image Analysis

**Figure 2:**
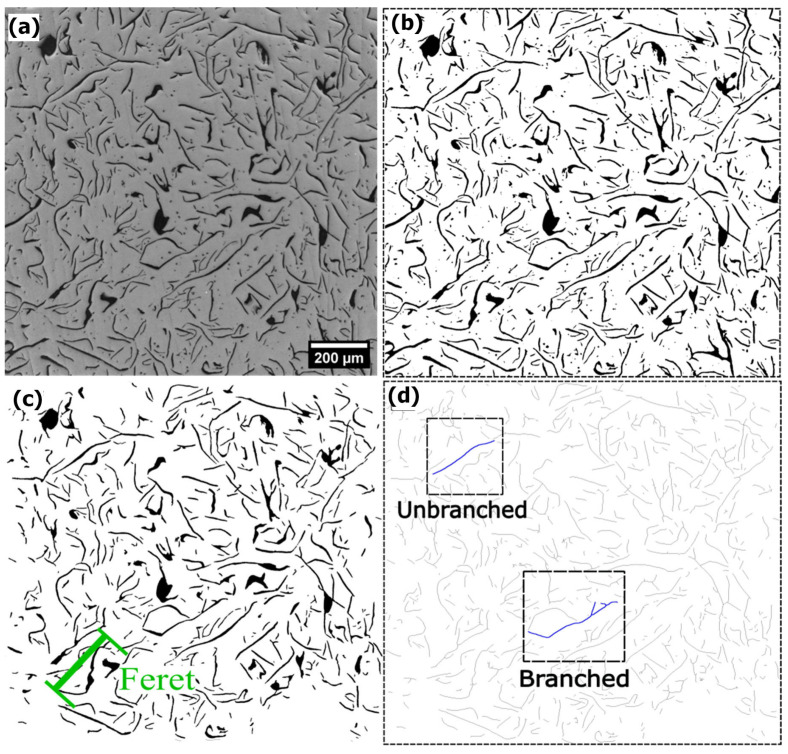
(**a**) original image without vignetting (100×), (**b**) binary image, (**c**) processed image, and (**d**) image resulting from the skeleton operation.

Six images at 100× were randomly taken from each metallographic sample. The image size is 2048 × 2048 pixels, corresponding to 1325 µm × 1325 µm at 100× magnification. The micrographs from unetched samples were taken in a Meiji Techno equipped with an electronic stage and the software Stage Manager 1.1, provided by DCI Microscopy. The uneven illumination, or vignetting, of the micrograph’s background was corrected using this software as well. This illumination correction is crucial for obtaining consistent results. In this study, the correction was made using a white reference image, i.e., a photograph from a polished surface without any microstructural features, taken with the same microscope setup used for the measurements. Figure 2a is an example of such corrected images. Image analysis was performed using ImageJ version 1.53 t software. The threshold for detecting the conversion of a grayscale image to a binary image corresponds to the default value provided by ImageJ, as shown in Figure 2b. The following operations were applied to the binary image to measure the Feret parameters. First, a fill holes operation was used, followed by “open,” which means erosion and dilation. Next, according to ISO 945-2 [31] recommendations, flakes that intersected the boundaries of the image frame and particles below 25 µm^2^ and 5 µm in Feret diameter were removed, resulting in the image shown in Figure 2c. The Feret parameter typically used in graphite flake characterization studies [11,48] represents the distance between the farthest ends of the flakes, as illustrated in Figure 2c. This value is considered in this work to be a reference value of previous studies reported by other authors [11,48].

**Figure 3:**
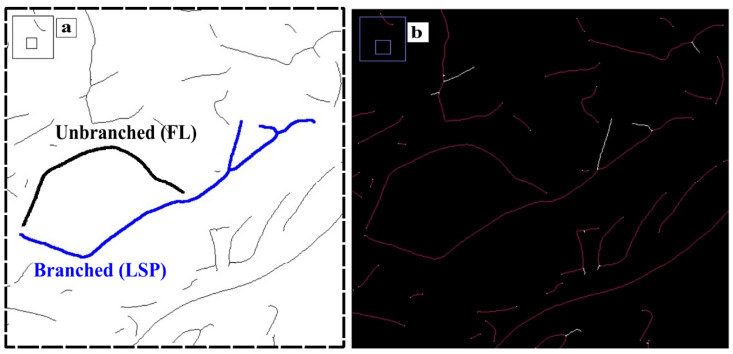
Pictures from inset “Branched” in Figure 2d. (**a**) Branched flake where LSP is measured and unbranched flake where its length is measured (FL), (**b**) Example of the Longest Shortest Path (LSP) of each flake, red lines, excluding the shorter branches (white lines).

Additionally, the skeletonization operation in ImageJ was explored to characterize the degree of branchedness in graphite flakes, as previously mentioned as a need [31]. This plugin was implemented by Arganda-Carreras [49] based on the work of Lee and colleagues [50], and it also performs the longest shortest path (LSP) calculations according to the work of Polder and colleagues [51]. ImageJ defines LSO as the longest (red lines in Figure 3b) among all the shortest paths connecting any two nodes (junctions or endpoints) within a connected component skeleton. The result of the skeletonization operation on the images is shown in Figure 2d. This operation reduces the particles to 1 pixel thickness, resulting in the “skeleton” of the particle. From the skeletonization operation, several characteristics of the flake can be obtained, such as whether it is branched or not, how many branches it has, and the LSP value. The value of flake’s length is denoted here as FL when the flake is not branched, as illustrated in Figure 3a. Finally, it should be noted that the skeletonization process allows for a more precise measurement of the maximum length of graphite flakes, LSP.

The graphite flakes from each sample were characterized using the average Feret, LSP, and FL values and their maximum values in each micrograph. The ratio of averages of Feret/LSP was calculated to estimate the straightness of the graphite flakes. A value of this parameter equal to 1 corresponds to a straight flake.

The branching percentage was estimated based on the ratio between branched and the sum of all flakes (branched and unbranched). Two branching percentages were calculated. A percentage of branched flakes as defined according to the following expression:

(2) $$\%B_{N}=\left(\frac{N_{BF}}{N_{TF}}\right)\times 100,$$

where $\%B_{N}$ is the percentage of branching based on the number of branched flakes, $N_{BF}$, and the total number of flakes within the measured fields, N_TF_. A weighted-length percentage of branched flakes was also calculated according to the following expression:

(3) $$\%B_{LSP}=\left(\frac{{\sum_{i=1}^{i=n}LSP}_{BF}}{\sum_{j=1}^{j=m}{LSP}_{TF}}\right)\times 100,$$

where $\%B_{LSP}$ is the branching percentage weighted according to the LSP value of flakes. ${\sum LSP}_{BF}$ is the sum of the LSP of branched flakes, and $\sum {LSP}_{TF}$ is the sum of the LSP of all flakes within the measured fields.

### 2.3. Eutectic Cell Counting

**Figure 4:**
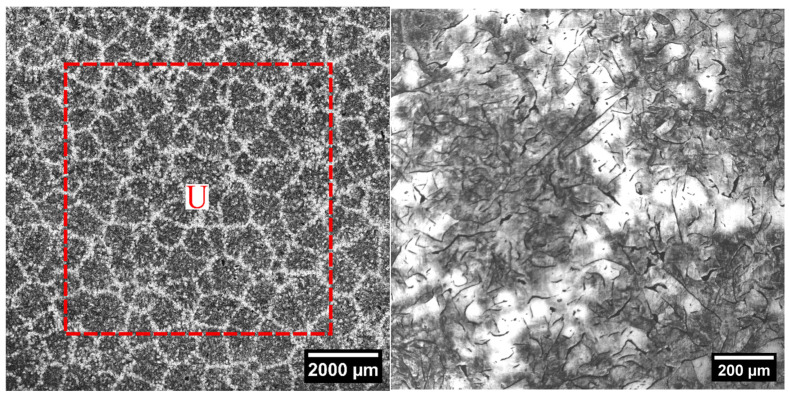
Eutectic cells shown by Stead reagent: (**left**) image displays the measured surface; (**right**) photograph shows eutectic cell details. Cell counts as per Equation (4). The red square (U) represents the area used for cell counting.

The samples were treated with Stead’s reagent to measure the number of eutectic cells. To prepare the reagent, 1000 mL of ethyl alcohol, 40 g of magnesium chloride, 10 g of cupric chloride, and 20 mL of hydrochloric acid were used, as specified by [52]. The eutectic cell count, N, was based on the following expression [52,53]:

(4) $$N=\frac{N_{i}+0.5N_{U}+1}{A},$$

where N_i_ is the number of complete cells within a square U, as shown in Figure 4, N_U_ is the number of cells that intersect with the square U borders (red dashed lines), excluding the cells at the corners, and A is the surface area of square U.

## 3. Results and Discussion

### 3.1. Sulfur and Manganese Concentrations

**Figure 5:**
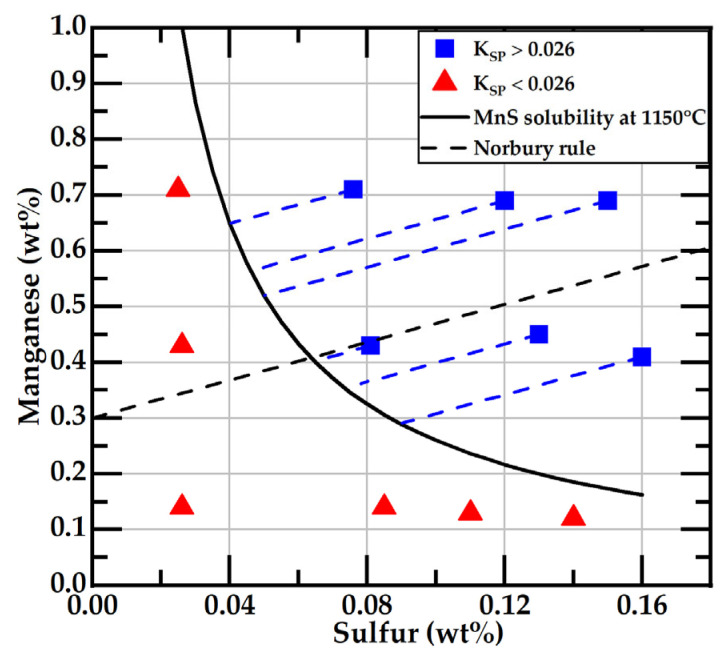
Manganese and sulfur content in the studied alloys. The black line represents the solubility curve at the eutectic temperature, K_SP_ = 0.026. The blue squares and red triangles are the data corresponding to the initial Mn and S concentrations. Blue dashed lines point to the free S content at 1150 °C for samples with Mn-S data above the solubility line.

Figure 5 displays the data of the initial concentrations of sulfur and manganese in the samples and the line corresponding to their solubility product K_SPE_ = 0.026 at 1150 °C. The procedure for obtaining this solubility product is in Appendix A. This value of the solubility product is slightly different from the one reported by Gundlach [11,12]. Figure 5 shows six compositions, represented by black squares, where the initial K_SP_ (wt%Mn × wt%S) product is below 0.026. According to the equilibrium conditions, without segregation of S and Mn during solidification, MnS cannot form in these samples. The free sulfur in the liquid metal at the eutectic solidification would correspond to the initial sulfur content in these compositions. The lowest sulfur content tested corresponds to three of these six compositions, while the other three have sulfur contents in the range of 0.08–0.14 wt%. In the six alloys represented by gray squares, whose K_SP_ product is above 0.026, the free sulfur content in the liquid metal during eutectic solidification could be located on the solubility curve, as illustrated in Figure 5 by white squares; it differs from the initial sulfur content.

Figure 5 shows that, with the designed experiment, both the initial sulfur content and the free sulfur at the eutectic temperature exhibit a wide range of variation, from ~0.026 wt% to ~0.15 wt%. Consequently, a significant variation in the morphology of the graphite flakes can be expected.

### 3.2. Classification of Graphite Morphology According to Standard A247-19 [26]

**Figure 6:**
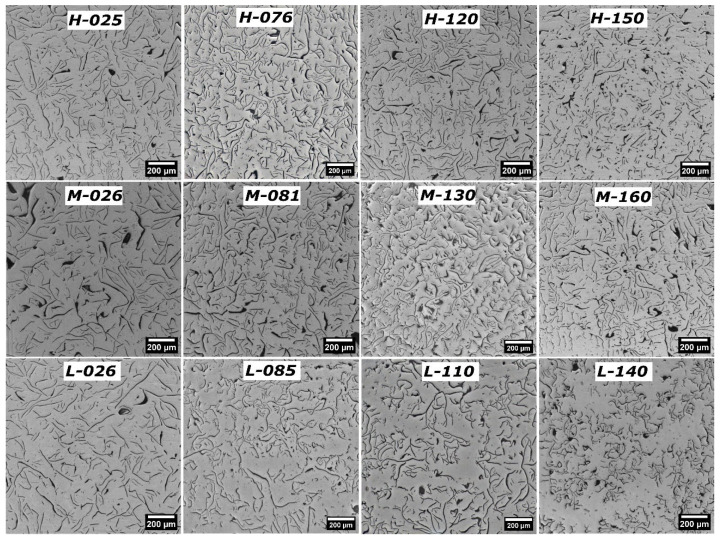
Graphite microstructure on the studied samples, organized according to their sulfur and manganese content. These micrographs show how sulfur and manganese levels affect graphite flake morphology when all other factors are constant. In the micrograph inserts, L, M, and H indicate low, medium, and high manganese levels; the numbers show sulfur content in thousandths of weight percent.

Figure 6 shows the typical micrographs of the microstructure present in the samples studied. The micrographs were located according to the distribution of compositions shown in Figure 5; the micrographs on the left and bottom correspond to the alloys whose solubility product K_SP_ is below 0.026. Upon comparing all the micrographs, a notable change in the morphology and distribution of graphite flakes is evident as a function of Mn and S contents. Samples with the lowest sulfur contents, specifically those with ~0.026 wt%, tend to exhibit the most uniformly distributed flake sizes, compared to samples with higher sulfur contents with the same manganese content. Graphite on samples with ~0.026 wt%S can be classified as type A. Samples with higher free-sulfur content in the liquid during eutectic solidification with a low manganese content, L-110, and L-140, present mainly a graphite distribution of type B. Most of the other samples present a graphite type A distribution, with a mixing of flake-size populations and, in some cases, with traces of type B. Table 2 shows the classification of the morphology and distribution of graphite flakes of all samples, following the A247-19 [30]) and ISO945-1 [27] standards.

The sample with the lowest contents of S and Mn, L-026, exhibits graphite flakes that are longer than those observed in the sample with the highest contents of these elements, H-150. According to the A247-19 standard [26], based on the maximum size of the flakes, the graphite flakes in the L-026 and H-150 samples are class two and three, respectively. Furthermore, it is worth noting that most of the small particles observed in the image of the H-150 sample are graphite, although a few correspond to MnS precipitates.

The visual classification based on the mentioned standards presents a difficulty in that, in addition to the mixture of types of flake distribution, there are cases in which flake sizes cannot be classified within a single class, as observed in sample H-150. Different flake sizes may lead to an error in assessing the type of flake distribution. For example, type A could be classed as type B in sample H-150.

As shown in Table 2 and observed in Figure 6, the graphite flake size class, based on the A247-19 standard [30], exhibits a trend with changes in S content at similar Mn contents. Comparing samples with 0.025 wt%S and 0.15 wt%S with the same Mn level, the flakes with smaller sizes (higher class) are present in specimens with high sulfur content; the H-150 has the shortest flakes observed among all these samples. This behavior is also observed in the Feret_max and Feret_avg values, as shown in Table 2. These data are further analyzed in detail.

### 3.3. Measurement of Graphite Flake Sizes

Table 2 also reports the Feret values of the flakes. For each sample, two Feret values are reported: Feret_max and Feret_avg. Feret_max corresponds to the average of six maximum Feret values, one value for each of the six micrographs, while Feret_avg is the average value of the Feret values of all the flakes measured. The value of Feret_max was used to determine the flake size classes according to the A247-19 standard [30].

**Figure 7:**
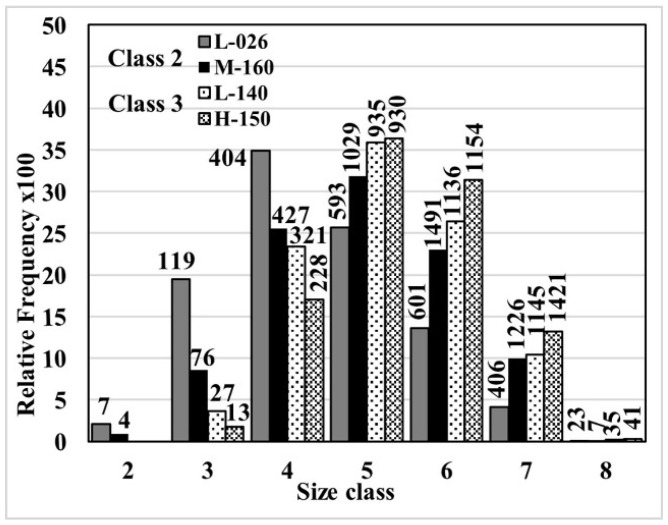
Relative frequency of sizes of the eight classes defined in the standard ASTM A247-19 [30]. The numbers above the bars indicate the total count of flakes of these sizes across the entire measured surface.

**Figure 8:**
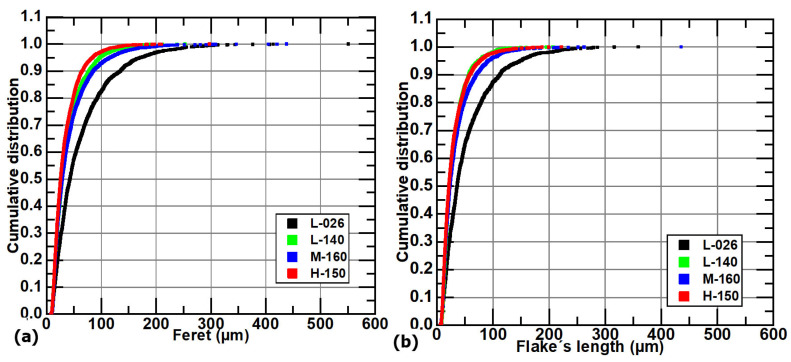
(**a**) Cumulative distribution of Feret and (**b**) FL values in samples L-026, L-140, M-160, and H-150.

Figure 7 presents the relative frequency histograms of the Feret diameter distribution for the L-026 and M-160 samples, class two flakes, and for the H-150 and L-140 samples, class three flakes. This figure shows that a small number of particles determine the class size. This criterion can be a drawback when a mixture of populations, such as sample M-160, exists, where only the proportion of larger particles determines the size classification, dismissing any influence of small particles. The consequences of such simplification can be observed in the cumulative distributions of the size of graphite lamellas, shown in Figure 8.

The cumulative distributions of the Feret diameters are plotted in Figure 8a, while the ones corresponding to the longest path on the flakes without branching are plotted in Figure 8b. Figure 8a shows that the L-026 sample exhibits the most extensive size distribution, with the largest flakes present throughout the entire size range. Remarkably, in Figure 8a, it is also observed that the cumulative distribution of sample M-160 (class two) is closer to the distributions of samples L-140 and H-150 (both class three) than that of sample L-026 (class two). If the mechanical properties or thermal conductivity are related to the graphite lamella size, some characteristics, such as average Feret values or those obtained from cumulative distribution plots, could be better descriptors of their representative size. Figure 8b shows the cumulative FL plots, corresponding to the unbranched flakes, which exhibit a similar trend to that observed in the cumulative curves shown in Figure 8a. The differences observed in those figures are mainly due to the branched flakes taken into account in the Feret measurement, Figure 8a, and not in FL plots, Figure 8b.

**Figure 9:**
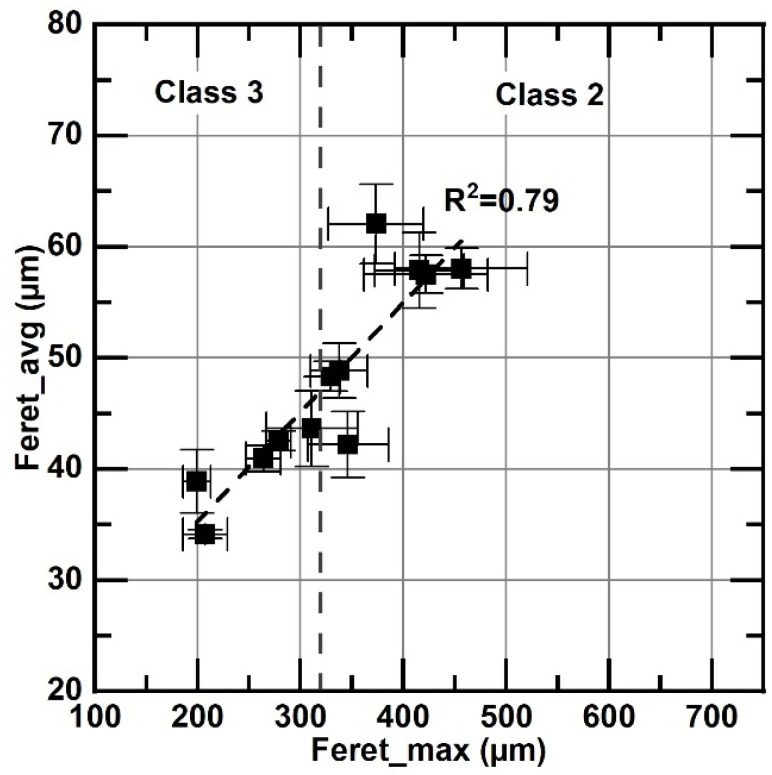
Correlation between Feret_max and Feret_avg. Error bars correspond to the standard error of the mean. The dashed line indicates the boundary between class 2 and class 3.

Instead of the size class, Feret_max is frequently used to characterize flake size [11,23]. The relationship between this parameter, flake size class, and Feret_avg is shown in Figure 9. This figure shows that, although the analyzed samples exhibit a wide range of Feret_max values, the information is reduced to only two size classes: class two and class three. Using size classes implies a loss in information resolution compared to Feret_max or Feret_avg values, which are also reasonably correlated (R^2^ = 0.79). These latter parameters are used in the following presentation of the results instead of class size.

**Figure 10:**
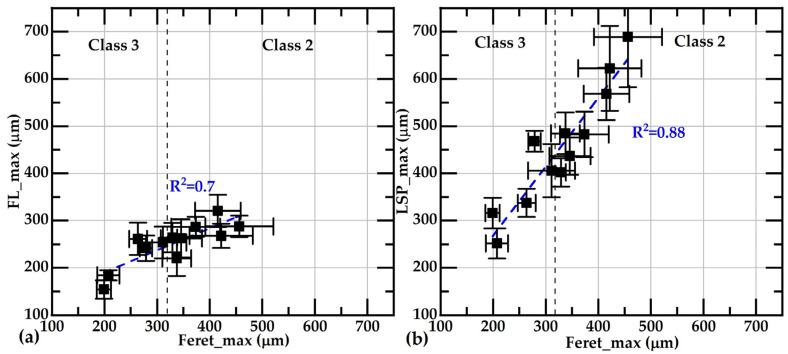
Correlations between Feret_max with FL_max (**a**), and LSP_max (**b**). Error bars correspond to the standard error of the mean. The dashed line in both subfigures indicates the boundary between classes 2 and 3. The subfigure on the left shows Feret_max and FL_max data, while the subfigure on the right displays Feret_max and LSP_max data.

**Figure 11:**
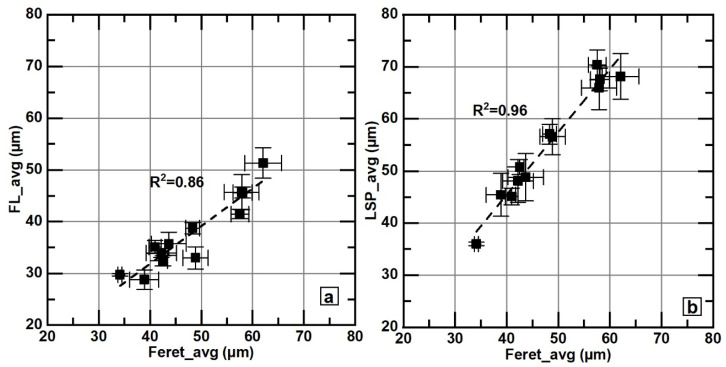
Correlations between average Feret values, FL_avg (**a**), and LSP_avg (**b**). Error bars correspond to the standard error of the mean. The subfigure on the left shows Feret_avg and FL_avg data, while the subfigure on the right displays Feret_avg and LSP_avg data.

The values of FL_max and LSP_max exhibit correlations of R2 = 0.7 and 0.88, respectively, with Feret_max; see Figure 10. The correlations between FL_avg and LSP_avg with Feret_avg are also reasonably good, as shown in Figure 11. The good correlations observed in all these figures indicate that these parameters are equivalent for comparative analysis purposes. These results also show that, to compare flake sizes between samples, Feret and the parameters related to the flake skeleton are equivalent; however, using size classification according to the ASTM A247-19 [30] standard implies a loss of data resolution.

Finally, it is worth noting that the values associated with LSP_max are larger than those associated with Feret_max. This difference originates from the fact that graphite flakes are not straight: the values of LSP and Feret would be equal in such flakes.

### 3.4. Effect of Mn and S on the Size of Graphite Flakes

Table 3 shows the set of all possible combinations between the morphological parameters of graphite flakes measured and the parameters associated with the Mn and S contents. All these combinations were graphically analyzed to elucidate any non-linear relationships. Additionally, both linear and quadratic correlations were estimated for these combinations. However, only the graphs showing data with better correlations are presented here, for simplicity. Table 3 presents the values of correlation coefficients and slopes obtained using a linear equation, because quadratic correlations show only slightly better values. The following graphs in this section illustrate the lines corresponding to linear and quadratic correlations.

In Table 3, it is notable that, in general, the correlation coefficient values are low; however, all parameters associated with the size of the flakes are better linearly correlated with the percentage of sulfur than with the other variables, manganese, Mn_ex_, or free S. Additionally, Table 3 indicates that the size parameters corresponding to the average values correlate more closely with sulfur. This better correlation can result from the averages considering many more measurements than the maximal values, i.e., the former has a more statistically solid base than the latter.

**Figure 12:**
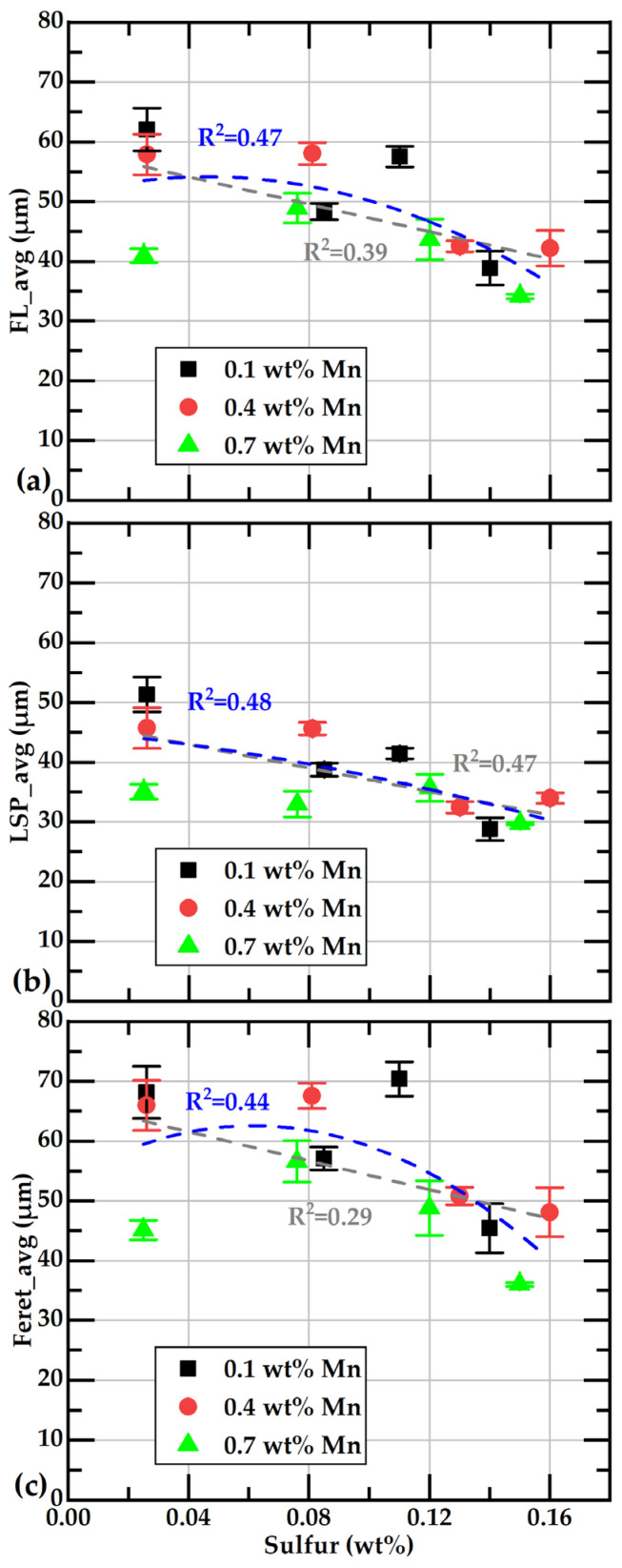
Effect of sulfur contents on the average parameter values associated with the size of graphite flakes. Subfigures (**a**–**c**) present the data for FL_avg, LSP_avg, and Feret_avg, respectively, as functions of the sulfur content.

Figure 12 illustrates the relationship between the different average values of the flake size and the percentage of sulfur, along with the corresponding lines for linear and quadratic correlation. It can be observed that, at each sulfur level, the smallest flakes are present at 0.7 wt%Mn, represented by dark squares. The reduction in flake size is more pronounced when the sulfur content exceeds 0.1 wt%, as indicated by the quadratic correlation lines. Other authors have also observed a decrease in graphite flake size as sulfur content increases [17,54].

Small flakes could decrease the UTS values [11]. The smaller Feret_avg values have been found in this study at higher Mn and sulfur concentrations. Sample H-150 is an example of such a material with small flakes. Interestingly, the free sulfur content of this sample at 1150 °C is 0.05 wt%, which is lower than the 0.14 wt% found in sample L-140. Both samples show small flakes. However, L-140 presents mainly a type B distribution of graphite flakes, while H-150 presents a type A distribution; the latter is more known for its better mechanical properties. Another difference between the two samples that can be observed in the corresponding micrographs in Figure 6 is the variation in branching presented by the graphite flakes, with L-140 exhibiting flakes with more branching. The branching data are presented in the following section.

**Figure 13:**
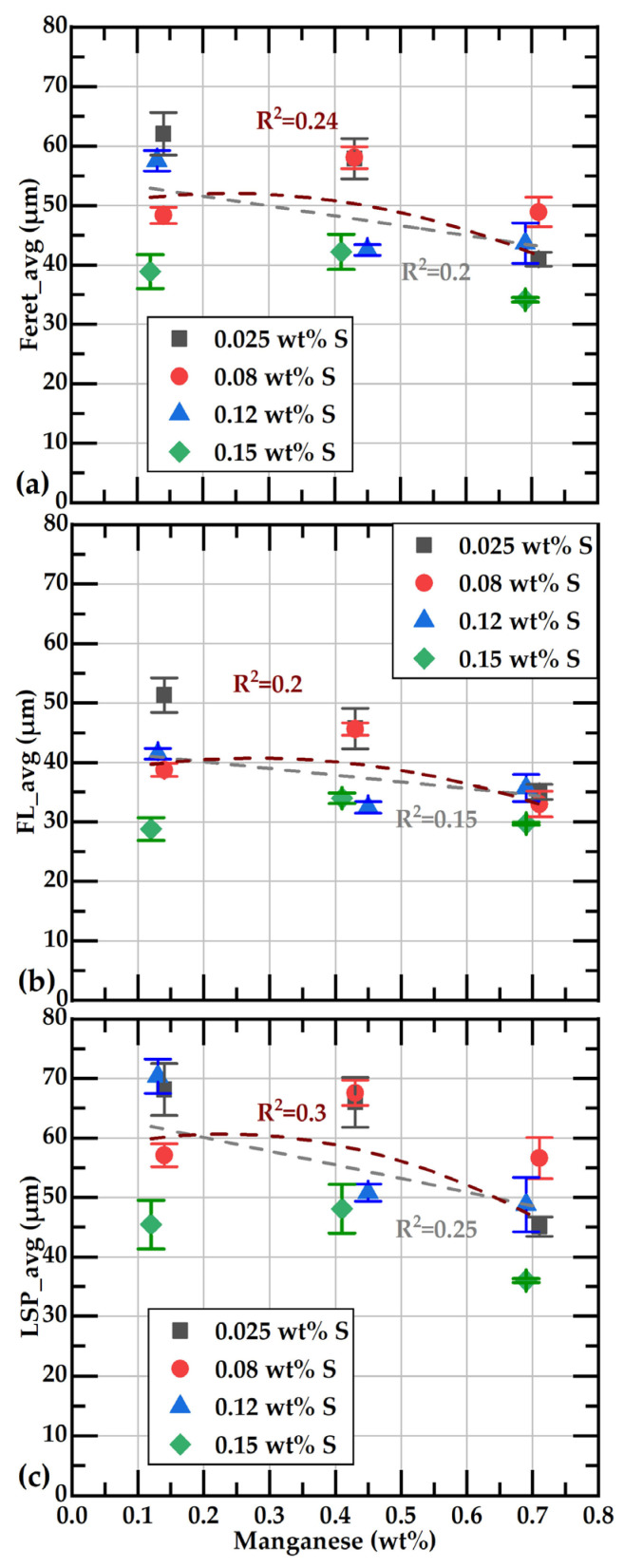
Effect of manganese contents on the average parameters’ values associated with sizes of graphite flakes. Subfigures (**a**–**c**) display the data for FL_avg, LSP_avg, and Feret_avg, respectively, as a function of the manganese content.

Figure 13 shows the relationship between the different average size values associated with flake size and the initial percentage of manganese. It can be observed that flake size values tend to decrease slightly with an increase in Mn content. It is also observed that, for samples with a given manganese content, those with the highest sulfur content have the smallest flakes. This trend is consistent with the reduction in average flake size with increasing sulfur content that was found by Gundlach et al. [12].

**Figure 14:**
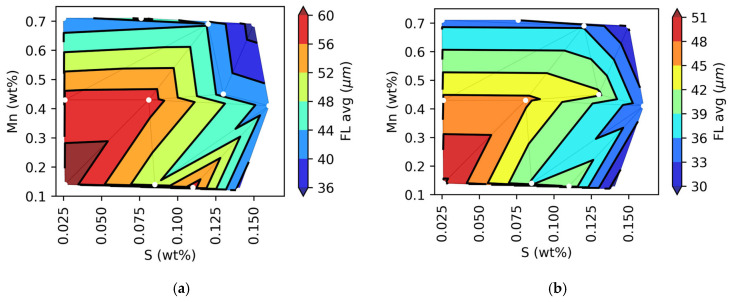
(**a**) Projection from 3D plot of the FL_avg or (**b**) Feret_avg parameters, with wt%Mn, and wt%S. White dots correspond to experimental data.

Although the correlation coefficients are small, a clear qualitative trend is easily observable when plotting S and Mn against FL_avg and Feret_avg values. Figure 14a,b shows the FL and Feret average values projected onto the S and Mn plane. These figures show that large flakes are obtained at low sulfur (<0.08 wt%) and manganese (<0.4 wt%) contents. They also show that FL and Feret average values are smaller and less dependent on Mn concentration when sulfur contents are higher than 0.12 wt%.

### 3.5. Effect of Mn and S on the Branching of Graphite Flakes

**Figure 15:**
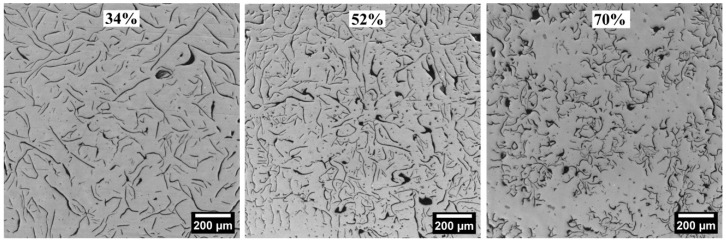
Representative micrographs with different percentages of branching, %B_LSP_.

Typical micrographs representing different branching values, %B_LSP_ (see Equation (3)) are shown in Figure 15. In these micrographs, it could be observed that the %B_LSP_ values around 34% correspond to A-type flake distribution, and the high value of %B_LSP_, 70%, is related to a B-type flake distribution. The values %B_N_ and %B_LSP_ were calculated using Equations (2) and (3), respectively.

**Figure 16:**
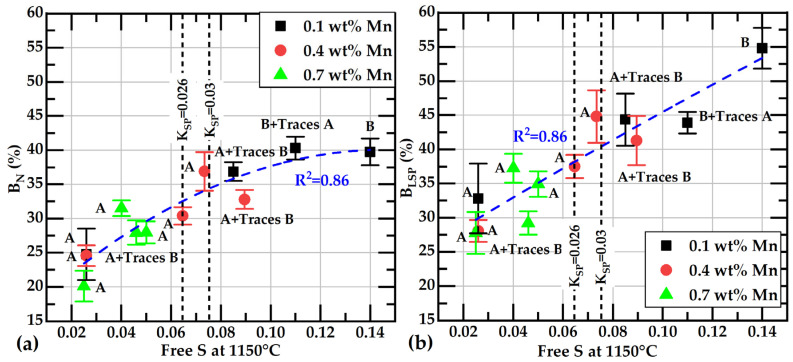
Branching values as a function of the free sulfur content at 1150 °C: (**a**) %B_N_ and (**b**) %B_LSP_. Blue dashed lines correspond to the quadratic regression of data. Vertical black dashed lines correspond to the K_SPE_ values of 0.026 and 0.03. Letters next to the data symbols, A and B, refer to graphite type distribution according to Table 2.

The presented data in Table 3 indicate that the highest correlation coefficients are associated with free S and %B_N_ or %B_LSP_, followed by the correlation coefficients of these branching parameters with Mn_ex_. Corresponding data for these parameters and free S, along with their respective quadratic correlations, are shown in Figure 16a,b. From comparing both figures, it can be observed that the values of %B_N_ are lower than those of %B_LSP_. Lower values of %B_N_ mean that the smaller-sized graphite flakes are more abundant than the larger-sized ones in our samples.

In the graphs of Figure 16, it can be observed that the appearance of B-type graphite distribution is related to free S values over 0.08 wt%. This value is close to that defined by a solubility constant, K_SPE_ = 0.03.

**Figure 17:**
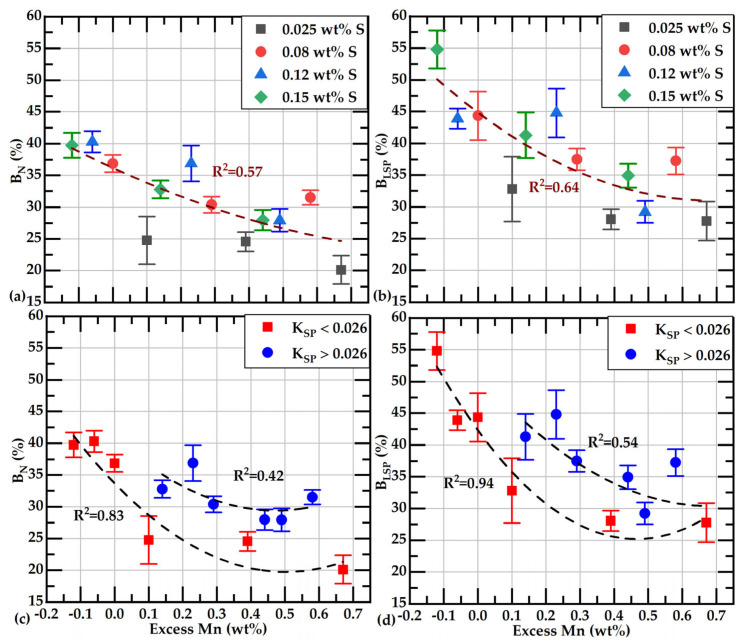
Branching values as a function of Mn_ex_. Dashed lines correspond to the quadratic regressions of the data. Subfigures (**a**,**b**) display B_N_(%) and B_LSP_(%) data grouped by sulfur content vs. %Mnex. Subfigures (**c**,**d**) show the same data vs. %Mnex, but grouped by Ksp value.

Figure 17 shows the branching values with their corresponding Mn_ex_ values. In Figure 17a,b, the branching values are further classified into four groups based on their initial sulfur content. These graphs show that in samples with a wt%S of 0.08 or higher, the branching decreases as Mn_ex_ increases; this behavior is less evident in samples with the lowest wt%S, 0.026%.

Because the effect of Mn_ex_ on branching may depend on whether Mn_ex_ modifies free S at 1150 °C during eutectic solidification, in Figure 17c,d, data are classified if their wt%Mn*wt%S product value is over or below the solubility product of MnS formation at 1150 °C, K_SPE_ = 0.026. These graphs show that both data groups exhibit the same trend of reducing the branching with increasing Mn_ex_, regardless of whether the K_SP_ value is below or above 0.026. However, data with K_SP_ > 0.026 show higher branching values than those with K_SP_ < 0.026. Interestingly, these graphs show that in cases where K_SP_ > 0.026, a Mn_ex_ of 0.3 is required to keep *B_n_* under 32% and *B*_LSP_ lower than 37.5%. Data with K_SP_ < 0.026 show the lowest branching values at a given Mn_ex_.

Based on visual appreciation, Fuller [8] noted that less branched graphite is present in samples with higher Mn_ex_ (at an initial 0.17 wt%S content), which agrees with the tendency observed here, based on the measured branching values.

**Figure 18:**
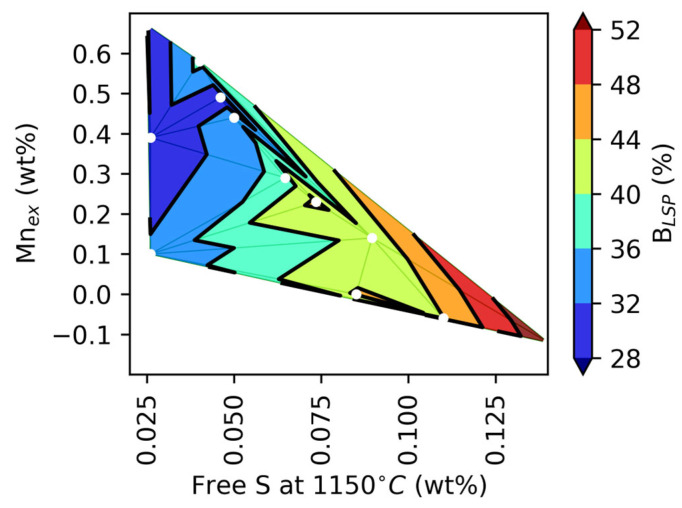
Projection based on 3D as a function of the %B_LSP_ parameter, wt%Mn, and wt%S. White dots correspond to experimental data.

Figure 18 summarizes the observed effect of Mn_ex_ and free S on % B_LSP_. It shows that when Mn_ex_ is lower than the value corresponding to Norbury’s rule (see Equation (1)), the tendency for branching increases.

### 3.6. Eutectic Cells Counting

**Figure 19:**
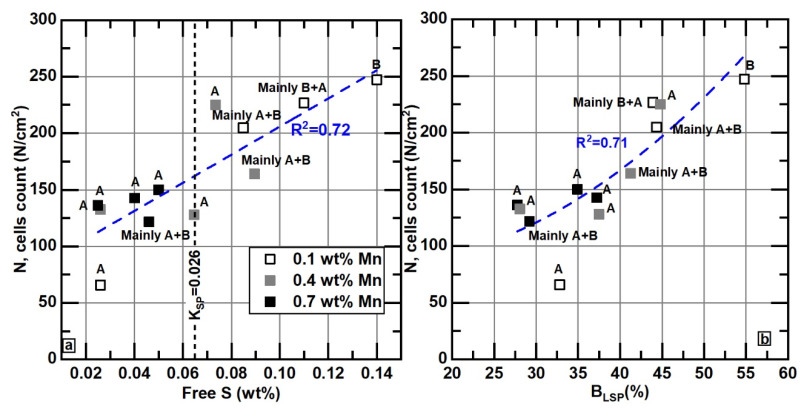
(**a**) Effect of free sulfur at 1150 °C on cell counting and its relationship with the %B_LSP_ parameter, (**b**) relationship between N and % B_LSP_. Dotted blue lines correspond to quadratic correlations. Letters A and B indicate the class of graphite distribution.

Figure 19a shows the data of the count of eutectic cells, N, as a function of free S. As can be seen, as the amount of free sulfur increases, the count of cells also increases. The N values were derived from Equation (4). Notably, the highest numbers of eutectic cells are present in samples with free S values over 0.07 wt%. Figure 19b represents the data of N as a function of % B_LSP_, showing that as % B_LSP_ increases, N also increases, and graphite with distribution type B tends to appear.

### 3.7. Summary of Main Effects of S and Mn on Branching, Flake’s Length, and Cell Count

**Figure 20:**
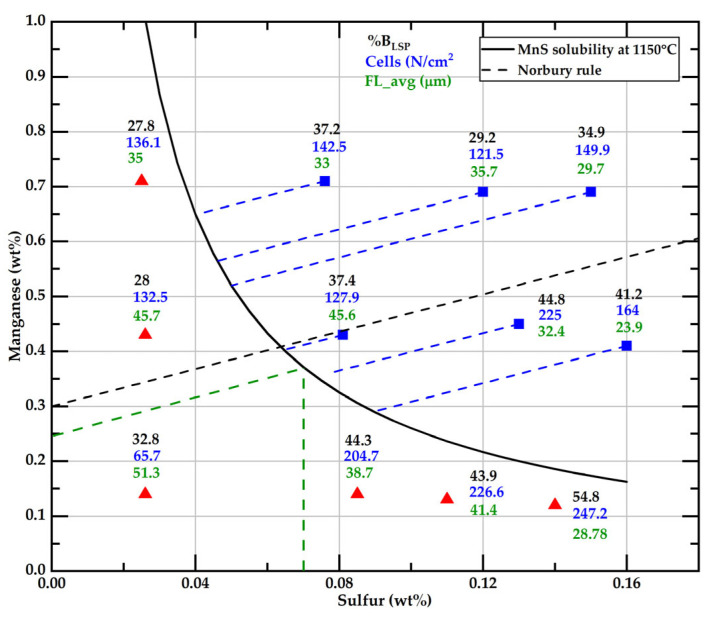
Data of the %B_LSP_, cell counting, and FL values as a function of initial contents of S and Mn. The solid line corresponds to a K_ps_ value of 0.026. Blue squares and red triangles indicate the initial contents of Mn and S, respectively. Blue dashed lines show the free S content at 1150 °C for samples with Mn-S data above the solubility line.

Figure 20 summarizes this work’s main measured microstructural features related to Mn, S, and free S. This figure shows that six samples, located above the solubility curve, have different values of free S per their corresponding Mn_ex_. One of these samples has a Mn_ex_ close to 0.3 wt%, corresponding to Norbury’s rule. Two of these samples have a Mn_ex_ under 0.3 wt%, which, together with the three samples with the lower Mn level and S higher than 0.08 wt%, form a group where the branching values are higher than 40%. The complementary group of samples shows branching values lower than 38%. The split limit over the solubility curve of both groups corresponds to a free S of 0.07 wt% and an Mn_ex_ of 0.25 wt% (green dashed lines). The split value is estimated to be 0.24 wt%Mn, from the free S value of 0.08 wt% proposed by Gundlach et al. [11]. Additionally, they observed that the maximum strength is obtained when the Mn and S contents are below the solubility curve and low sulfur levels are present. In such conditions, in our samples with wt%S below 0.04% the percentage of B_LSP_ is under 33%. This finding suggests that lower branching could be suitable for improving mechanical properties, and to achieve that morphological characteristic, low sulfur contents are needed. This idea needs to be tested by further research.

The data group with %B_LSP_ > 40% also corresponds to samples with the highest cell count compared to the group with %B_LSP_ < 38%. That observation agrees with Fuller’s observation, who found that the count of eutectic cells increased with an increase in initial sulfur content. Fuller attributed this effect to a change in the graphite growth kinetics with sulfur.

MnS is generally seen as a compound that assists graphite inoculation [6,37,55]. Supporting this idea, the lowest eutectic cell count was observed in the sample with about 0.026 wt% sulfur and 0.14 wt% Mn, where MnS likely does not form before the eutectic onset. However, this study also found the highest cell counts in three samples where MnS also does not form before eutectic onset, specifically when sulfur exceeds 0.08 wt% and Mn is below 0.14%. The high cell count in these samples could be a consequence of the effect of dissolved sulfur on the eutectic growth kinetics, also controlled by the Mn_ex_. Elucidating which of these two effects of Mn is predominant requires further research. Measuring the proportion of branched graphite flakes can be essential to achieve this goal.

**Figure 21:**
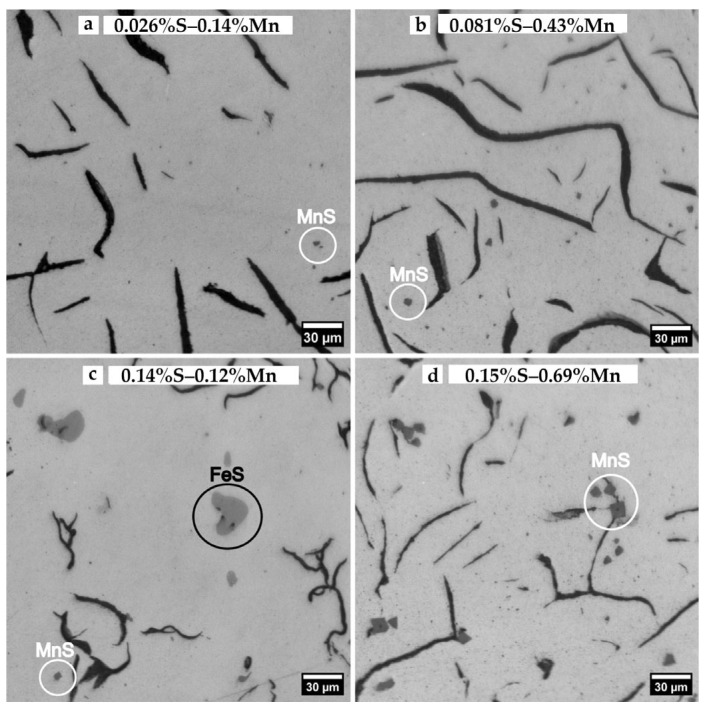
MnS precipitates as observed on samples with different Mn and S contents. Micrographs (**a**,**c**) show samples where the wt%S × wt%Mn (K_sp_) is less than 0.026, where MnS formation is not estimated before the eutectic reaction. Micrographs (**b**,**d**) show samples with K_sp_ values greater than 0.026, where MnS forms prior to the eutectic reaction. In the sample with 0.14 wt%S and 0.12 wt%Mn, some FeS particles were also observed. MnS precipitates are darker and more faceted than FeS. The micrographs were taken at 500×.

It should be mentioned that particles of MnS are observed in all samples, even in the samples where (wt%S × wt%Mn) is below the solubility curve, as illustrated in Figure 21. In these samples, MnS precipitates are smaller and less numerous than in the other samples, and even FeS is observed in the sample with 0.14 wt%S and 0.12 wt%Mn. In these samples, MnS precipitates could form due to the microsegregation of S and Mn, which is not accounted for in the thermodynamic estimation. The proportion of MnS precipitates observed in contact with the graphite is significantly higher in samples above wt%S × wt%Mn = 0.026 (see Figure 21d), which has been considered in the bibliography to be evidence that MnS can enhance graphite nucleation.

## 4. Conclusions

Hypoeutectic cast irons with a similar composition, except the Mn and S contents, were poured in keel blocks using the same procedure of melting, inoculation, and pouring. The following conclusions were obtained from the microstructural characterization of samples taken at the same location in keel blocks obtained from this work.

- It was found that the classification of flake size by class, based on Feret_max, according to ASTM A247-19 [30], implies a loss of important information about the effect of Mn and S on the length of graphite flakes. Samples of the same size class could have a noticeable difference in the flake’s length; for example, samples L-026 and M-160 show very different Feret_avg values, 62 and 42 μm, but both are class two. The frequency histograms and the cumulative distribution of Feret values show that average Feret values are better descriptors of the whole distribution of the flake’s size.
- These results also show that for flake size comparison between samples, all parameters tested here, Feret_avg, Feret_max, FL_max, FL_avg, LSP_avg and LSP_max, are equivalent.
- The larger flake sizes were observed in the samples with the lower content in both S and Mn. The effect of Mn content on Feret_avg or FL_avg values is less significant in the samples with sulfur contents over 0.12 wt% than in samples under 0.12 wt%S. At the highest sulfur contents, the size of the graphite flakes is the smallest at all Mn concentrations.
- A new approach is proposed to quantify the branching of graphite flakes of cast irons. With this technique, the percentage of branched flakes, i.e., flakes having one or more branches in the overall population of flakes, can be quantitatively obtained. The percentage of branched flakes was calculated in the basis of the number of flakes, %B_N_, or on a weighted basis, %B_LSP_, considering the length of the flakes. These parameters exhibit an excellent correlation with the free S of the liquid metal estimated at 1150 °C. The branching of flakes is diminished when the contents of free sulfur decrease.
- However, the free S is not well correlated with the flakes’ size_._ Then, with a similar size, the graphite flakes could have different branching values. It was observed that the longest and lowest-branched graphite flakes were obtained from the casting with 0.026 wt%S. Free sulfur and sulfur content have the same value in those castings.
- In our samples, the branching parameter values, %B_N_ or %B_LSP_, are associated with the type of graphite distribution, as determined by visual inspection. Type B graphite exhibits greater branching compared to Type A graphite. The broader applicability of this observation should be validated through additional research.
- It was observed that when free S increases, the number of eutectic cells, the branching of the graphite flakes, and the presence of Type B graphite increase.

## Figures and Tables

**Table 1:** Chemical composition of samples and values of “wt%Mn_ex_”, “wt%Mn·wt%S” and free S (wt%).

Sample	wt% C	wt% Si	wt% CE	wt% S	wt% Mn	wt% Cu	wt% P	Excess Mn (wt% Mn_ex_)	K_sp_	Free S (wt%)
L-026	3.31	2.03	3.99	0.026	0.14	0.84	0.011	0.1	0.004	0.026
L-085	3.3	2.05	3.99	0.085	0.14	0.83	0.013	0	0.012	0.085
L-110	3.3	2.03	3.98	0.11	0.13	0.84	0.013	−0.06	0.014	0.110
L-140	3.32	2.01	4.00	0.14	0.12	0.85	0.015	−0.12	0.017	0.140
M-026	3.32	2.13	4.04	0.026	0.43	0.83	0.015	0.39	0.011	0.026
M-081	3.35	2.11	4.06	0.081	0.43	0.84	0.014	0.29	0.035	0.065
M-130	3.35	2.22	4.10	0.13	0.45	0.82	0.015	0.23	0.059	0.073
M-160	3.38	2.11	4.0	0.16	0.41	0.85	0.016	0.14	0.066	0.090
H-025	3.27	2.00	3.94	0.025	0.71	0.85	0.018	0.67	0.018	0.025
H-076	3.28	2.04	3.97	0.076	0.71	0.85	0.020	0.58	0.054	0.040
H-120	3.31	2.03	3.99	0.12	0.69	0.86	0.019	0.49	0.083	0.046
H-150	3.30	1.99	3.97	0.15	0.69	0.85	0.022	0.44	0.104	0.050

**Table 2:** Classification of graphite based on size and type according to the A247-19 [26] standard. The contents of Mn, S and K_sp_ and Mn_ex_ values from Table 1 are presented here to facilitate the analysis of the results. Graphite type is related to flake distribution in the matrix. Size of flakes is characterized by three parameters: size class and values of Feret_avg or Feret_max.

Sample	S (%wt)	Mn (%wt)	Mn_ex_ (%wt)	“K_sp_”	Size Class	Graphite Type (Visual Analysis)	Feret_avg (µm)	Feret_max (µm)
L-026	0.026	0.14	0.1	0.004	2	Type A	62	373.4
L-085	0.085	0.14	0	0.012	2	Type A+ traces of type B	48.3	328.9
L-110	0.11	0.13	−0.06	0.014	2	Type B, traces of type A	57.5	421.8
L-140	0.14	0.12	−0.12	0.017	3	Type B	38.9	198.9
M-026	0.026	0.43	0.39	0.011	2	Type A	57.9	415.5
M-081	0.081	0.43	0.29	0.035	2	Type A	58	456.3
M-130	0.13	0.45	0.23	0.059	3	Type A	42.5	278.7
M-160	0.16	0.41	0.14	0.066	2	Type A, traces of type B	42.2	346.1
H-025	0.025	0.71	0.67	0.018	3	Type A	41	263.8
H-076	0.076	0.71	0.58	0.054	2	Type A	48.9	337.4
H-120	0.12	0.69	0.49	0.083	3	Type A, traces of type B	43.7	310.9
H-150	0.15	0.69	0.44	0.104	3	Type A	34.1	207.2

**Table 3:** Linear correlation parameters between the different microstructural parameters measured and the composition variables studied in this work.

	x	Sulfur		Manganese		Mn_ex_		Free S	
y									
		R^2^	m	R^2^	m	R^2^	m	R^2^	m
Feret avg		0.39	−114.2	0.2	−16.5	0.05	−7.6	0.05	−56.5
Feret max		0.18	−707.1	0.06	−83	0.01	−31.4	0.036	−423.8
LSP avg		0.29	−120.9	0.25	−23.04	0.09	−13.1	0.007	−25.8
LSP max		0.1	−823.1	0.07	−138.2	0.03	−74.5	0.002	−133.9
FL avg		0.47	−98.5	0.15	−11.3	0.02	−3.9	0.15	−74.3
FL max		0.39	−580	0.01	−18.8	0.01	19.5	0.23	−607.4
%B_LSP_		0.36	99.4	0.38	−20.7	0.6	−24	0.86	207.4
%B_N_		0.48	93.7	0.3	−15.2	0.57	−19.4	0.83	165.7
Cells count		0.32	619.8	0.15	−85.8	0.3	−115.4	0.72	1243.7

## Data Availability

All original data for this research were included in this article. Further inquiries can be directed to the corresponding author.

## Abbreviations

The following abbreviations are used in this manuscript:

CE	Carbon Equivalent
UTS	Ultimate Tensile Strength
%Mn_ex_	Excess of manganese defined in Equation (1)
K_SPE_	Equilibrium solubility product of M and S in liquid iron at 1150 °C
K_SP_	Solubility product, (wt%Mn) (wt%S), corresponding to initial concentrations
Feret_max	Average of six maximum Feret values for sample, one value for micrograph
Feret_avg	Average value of the Feret of all the flakes measured.
FL	Length of an unbranched flake
FL_max	Average of six maximum FL values for sample, one value for micrograph
FL_avg	Average value of the FL of all the flakes measured
LSP	Longest Shortest Path in a flake
LSP_max	Average of six maximum LSP values for sample, one value for micrograph
LSP_avg	Average value of the LSP of all the flakes measured
$\%B_{N}$	Percentage of branched flakes based on the number of flakes
$\%B_{LSP}$	Percentage of branched flakes weighted according to the value of flakes
N	Eutectic cell count/cm^2^

## Appendix A

### Calculus of the Sulfur Concentration in Liquid Metal at 1150 °C, Free S

The free energy of the MnS formation from dissolved Mn and S in liquid iron, Mn_(%)_ and S_(%)_, was obtained from the data shown in Table A1.

**Table A1:** Data of free energy formation of MnS. (s) solid, (g) gas, (l) liquid, (%) weight per cent in Fe.

	Reaction	$∆G^{o}=∆H^{o}-∆S^{o}(\frac{J}{mol})$	Ref.
1	${Mn}_{\left(s\right)}+\frac{1}{2}S_{2(g)}={MnS}_{\left(s\right)}$	−277,900 + 64 T	[56]
2	${Mn}_{\left(l\right)}={Mn}_{\left(s\right)}$	−14,600 +9.6 T	[56]
3	${Mn}_{\left(\%\right)}={Mn}_{\left(l\right)}$	−4086.31 + 38.15 T	[57]
4	$S_{\left(\%\right)}=\frac{1}{2}S_{2(g)}$	135,149.9 − 23.44 T	[57]
5	${Mn}_{\left(\%\right)}+S_{\left(\%\right)}={MnS}_{\left(s\right)}$	−161,436.4 + 88.3 T	

${∆G}_{{MnS}_{\left(s\right)}}^{o}=-RT\;lnK=RTln(f_{s}wt\%S)(f_{Mn}wt\%Mn).$

An estimate of the Henrian coefficient values at 1150 °C was obtained from the interaction parameters, $e_{i}^{j}$, provided by Sigworth [57]. These interaction parameters take into account the effect of element *j* on *f_i_*, according to the expression:

$$logf_{i}=\sum_{j=2}^{n}e_{i}^{j}(wt\%j)$$

$$logf_{Mn}=e_{Mn}^{C}wt\%C+e_{Mn}^{Si}wt\%Si+e_{Mn}^{P}wt\%P+e_{Mn}^{S}wt\%S+e_{Mn}^{Cu}wt\%Cu\ldots$$

$${logf}_{S}=e_{S}^{C}wt\%C+e_{S}^{Si}wt\%Si+e_{S}^{P}wt\%P+e_{S}^{Mn}wt\%Mn+e_{S}^{Cu}wt\%Cu\ldots$$

**Table A2:** Interaction coefficients $e_{j}^{i}$ of the solutes S and Mn in liquid iron at 1600 °C.

		*j*				
*i*	C	Si	P	Cu	S	Mn
S	0.11	0.063	0.29	−0.0084	-	−0.026
Mn	−0.07	0	−0.0035	0	−0.048	-

Performing this calculation for the average composition presented in Table 1 obtained $f_{Mn}=0.577$ and $f_{s}=3.24$.

## References

[1] (2022). Standard Specification for Gray Iron Castings.

[2] Craig D.B., Hornung M.J., McCluhan T.K. (1998). Gray Iron. Casting.

[3] Li Q., Zhang Y., Zhang Y., Liu H., Ren H., Zhong Y., Huang X., Huang W. (2020). Influence of Sn and Nb Additions on the Microstructure and Wear Characteristics of a Gray Cast Iron. Appl. Phys. A.

[4] Lacaze J., Sertucha J. (2016). Effect of Cu, Mn and Sn on Pearlite Growth Kinetics in as-Cast Ductile Irons. Int. J. Cast Met. Res..

[5] Upadhyay S., Saxena K.K. (2020). Effect of Cu and Mo Addition on Mechanical Properties and Microstructure of Grey Cast Iron: An Overview. Mater. Today: Proc..

[6] Goodrich G.M., Oakwood T.G., Gundlach R.B. (2003). Manganese, Sulfur and Manganese-Sulfur Ratio Effects in Gray Cast Iron. AFS Trans..

[7] Norbury A.L. (1929). Manganese in Cast Iron.

[8] Fuller A.G. (1986). Effect of Manganese and Sulfur on Mechanical Properties and Structure of Flake Graphite Cast Irons. AFS Trans..

[9] Alderson A. (1983). The Influence of Manganese and Sulfur on the Strtucture and Mechanical Properties of Grey Cast Iron. Br. Foundrym..

[10] Muhmond H.M., Fredriksson H. (2013). Relationship between Inoculants and the Morphologies of MnS and Graphite in Gray Cast Iron. Metall. Mater. Trans. B.

[11] Gundlach R. (2015). Influence of Mn and S on the Properties of Cast Iron Part III—Testing and Analysis. Int. J. Met..

[12] Gundlach R. (2018). Influence of Mn and S on the Microstructure of Cast Iron. AFS Trans..

[13] Srivastava R., Singh B., Saxena K.K. (2020). Influence of S and Mn on Mechanical Properties and Microstructure of Grey Cast Iron: An Overview. Mater. Today Proc..

[14] Mampaey F. (1981). The Manganese–Sulphur Ratio in Grey Iron. Fonderie Belg..

[15] Lacaze J., Connétable D., Castro-Román M.J. (2019). Effects of Impurities on Graphite Shape during Solidification of Spheroidal Graphite Cast Ions. Materialia.

[16] Mills K. (1985). ASM Metals Handbook.

[17] Nakae H., Shin H. (2001). Effect of Graphite Morphology on Tensile Properties of Flake Graphite Cast Iron. Mater. Trans..

[18] Chen Y., Xue Z., Song S., Cromarty R., Zhou X. (2023). Evolution of Microstructure and High Temperature Tensile Strength of Gray Cast Iron HT250: The Role of Molybdenum. Mater. Sci. Eng. A.

[19] Du S., Chen C., Chen R., Wang Q., Cui X., Song Q. (2025). Influence of Casting Materials on the Microstructure and Mechanical Properties of Gray Cast Iron for Cylinder Liners. Inter Met..

[20] Uchimoto T., Takagi T., Abe T. (2010). Electromagnetic Nondestructive Evaluation of Graphite Structures in Flake Graphite Cast Iron. Mater. Trans..

[21] Holmgren D., Svensson I.L. (2005). Thermal Conductivity–Structure Relationships in Grey Cast Iron. Int. J. Cast Met. Res..

[22] Wang G., Li Y. (2020). Thermal Conductivity of Cast Iron -A Review. China Foundry.

[23] Hecht R.L., Dinwiddie R.B., Wang H. (1999). The Effect of Graphite Flake Morphology on the Thermal Diffusivity of Gray Cast Irons Used for Automotive Brake Discs. J. Mater. Sci..

[24] Tewary U., Paul D., Mehtani H.K., Bhagavath S., Alankar A., Mohapatra G., Sahay S.S., Panwar A.S., Karagadde S., Samajdar I. (2022). The Origin of Graphite Morphology in Cast Iron. Acta Mater..

[25] Amini S., Abbaschian R. (2013). Nucleation and Growth Kinetics of Graphene Layers from a Molten Phase. Carbon.

[26] (2019). Standard Test Method for Evaluating the Microstructure of Graphite in Iron Castings.

[27] (2019). Microstructure of Cast Irons—Part 1: Graphite Classification by Visual Analysis.

[28] Cree J.W., Hoover A.M., Pelland B.C., Pearson G.R., Cruse J.L., Naik N.A. (2025). Statistical Analysis of Effects of Boron and Titanium, Plus Plain Thermal Analysis (TA) Cup Type on Quantified Image Analysis Metallography, Ultrasonic Velocity and Mechanical Properties of Gray Iron. Inter Met..

[29] Khuntrakool C., Janudom S., Muangjunburee P., Yodjan A., Mahathaninwong N., Chucheep T. (2025). Control of Graphite Structure in High Phosphorus Grey Cast Iron Brake Shoes through Ferro-Silicon Inoculant. Inter Met..

[30] (1967). Standard Test Method for Evaluating the Microstructure of Graphite in Iron Castings.

[31] (2011). Microstructure of Cast Irons—Part 2: Graphite Classification by Image Analysis.

[32] Chen T., Wang C., Yan R., Li F., Wang J., Wang J. (2024). Study on Friction and Wear Behavior of Gray Cast Iron with Different Carbon Content at Different Temperatures. Mater. Res. Express.

[33] Ji L., Du X., Sun Y., Zhu T., Feng J. (2024). Effect of Solidification Rate on Microstructure and Mechanical Characteristic of Gray Cast Iron. Inter Met..

[34] Li Z., Chen R., Wang Q., Chen C., Zhang Y., Song Q. (2025). Effect of Nb Addition on Compact Microstructure and Mechanical Properties of Gray Cast Iron for Cylinder Liner. Inter Met..

[35] Barlow T.E., Lorig C.H. (1946). Gray Cast Iron Tensile Strength, Brinell Hardness and Composition Relationships. AFS Trans..

[36] Goodrich G.M. (1997). Cast Iron Quality Control Committee 5J Report Cast Iron Microstructure Anomalies and Their Causes. AFS Trans..

[37] Gundlach R.B. (2014). Influence of Mn and S on the Mechanical Properties of Gray Cast Iron: Part I—Historical Perspective. AFS Trans..

[38] Prakash P., Mytri V., Hiremath P. (2011). Fuzzy Rule Based Classification and Quantification of Graphite Inclusions from Microstructure Images of Cast Iron. Microsc. Microanal..

[39] Friess J., Sonntag U., Steller I., Bührig-Polaczek A. (2020). From Individual Graphite Assignment to an Improved Digital Image Analysis of Ductile Iron. Inter Met..

[40] Huang W., Su Z.-Y., Wang C.-S., Yeh M., Chou J.-H. (2022). Graphite Classification of Gray Cast Iron in Metallographic via a Deep Learning Approach. J. Internet Technol..

[41] Germain L., Sertucha J., Hazotte A., Lacaze J. (2024). Classification of Graphite Particles in Metallographic Images of Cast Irons—Quantitative Image Analysis versus Deep Learning. Mater. Charact..

[42] Lin C., Chen C., Wang W., Pei X., Hu W., Su S. (2024). Graphite Particle Segmentation Method for Spheroidal Graphite Cast Iron Based on Improved DeepLabv3+. Inter Met..

[43] Waqas M., Sohail M., Anwer S., Kazmi M., Raza B. (2025). Microstructural Characterization and Analysis of Gray Cast Iron Material Using Deep Learning-Based Object Detection and Localization Techniques. Int. J. Comp. Mat. Sci. Eng..

[44] Chuang C., Singh D., Kenesei P., Almer J., Hryn J., Huff R. (2015). 3D Quantitative Analysis of Graphite Morphology in High Strength Cast Iron by High-Energy X-Ray Tomography. Scr. Mater..

[45] Lemiasheuski A., Kranzmann A., Pfennig A. (2024). Challenges and Possibilities of the Manual Metallographic Serial Sectioning Process Using the Example of a Quantitative Microstructural Analysis of Graphite in Cast Iron. Pract. Metallogr..

[46] Domeij B., Hernando J.C., Diószegi A. (2018). Size Distribution of Graphite Nodules in Hypereutectic Cast Irons of Varying Nodularity. Metall. Mater. Trans. B.

[47] (2019). Fundición. Fundición de Grafito Esferoidal.

[48] Griffin R.D., Scarber P., Janowski G.M., Bates C.E. (1996). Quantitative Characterization of Graphite in Gray Iron. AFS Trans..

[49] Arganda-Carreras I., Fernández-González R., Muñoz-Barrutia A., Ortiz-De-Solorzano C. (2010). 3D Reconstruction of Histological Sections: Application to Mammary Gland Tissue. Microsc. Res. Tech..

[50] Lee T.-C., Kashyap R.L., Chu C.-N. (1994). Building Skeleton Models via 3-D Medial Surface Axis Thinning Algorithms. CVGIP Graph. Models Image Process..

[51] Polder G., Hovens H.L.E., Zweers A.J. Measuring Shoot Length of Submerged Aquatic Plants Using Graph Analysis. Proceedings of the ImageJ User and Developer Conference 2010.

[52] Fras E., Górny M., Kapturkiewicz W., Lopez H. (2007). Eutectic Cell and Nodule Count in Cast Irons. Int. J. Cast Met. Res..

[53] Ryś J. (1995). Stereology of Materials.

[54] Moumeni E. (2013). Solidification of Cast Iron-A Study on the Effect of Microalloy Elements on Cast Iron. Ph.D. Thesis.

[55] Moumeni E., Stefanescu D.M., Tiedje N.S., Larrañaga P., Hattel J.H. (2013). Investigation on the Effect of Sulfur and Titanium on the Microstructure of Lamellar Graphite Iron. Metall. Mater. Trans. A.

[56] Fruehan R.J. (1998). The Making, Shaping and Treating of Steel.

[57] Sigworth G.K., Elliott J.F. (1974). The Thermodynamics of Liquid Dilute Iron Alloys. Met. Sci..

